# Safety and efficacy of belt-type electrical stimulation for preventing disuse syndrome in elderly hemodialysis patients: a pilot study

**DOI:** 10.3389/fresc.2025.1559077

**Published:** 2025-07-17

**Authors:** Misa Miura, Shigeru Owada, Osamu Ito, Masahiro Kohzuki

**Affiliations:** ^1^ Department of Physical Therapy, Faculty of Health Sciences, Tsukuba University of Technology, Tsukuba, Japan; ^2^ Asao Clinic, Kawasaki, Japan; ^3^ Division of General Medicine and Rehabilitation, Faculty of Medicine, Tohoku Medical Pharmaceutical University, Sendai, Japan; ^4^ Yamagata Prefectural University of Health Sciences, Yamagata, Japan

**Keywords:** elderly hemodialysis patients, belt-type electrical stimulation, safety, physical function, body composition, skeletal muscle mass hemodialysis, elderly, electrical stimulation

## Abstract

**Background:**

Elderly hemodialysis (HD) patients frequently experience reduced physical activity due to treatment-related immobility and fatigue, leading to accelerated functional decline. While exercise therapy is beneficial, adherence remains challenging among frail elderly patients. Belt-type electrical stimulation (B-SES) presents a potential alternative, though evidence for its safety and efficacy in this population is limited.

**Methods:**

In this 12-week prospective intervention study, eight frail maintenance HD patients (mean age 75.5 ± 0.9 years) received B-SES therapy during HD sessions. Outcomes were assessed through physical function measures, biochemical markers, quality of life indices, and body composition analysis using multiple imaging modalities (BIA, CT, ^1^H-MRS).

**Results:**

Five participants completed the intervention, with three withdrawals unrelated to the treatment. Physical function measures showed a trend toward improvement without elevation in inflammatory markers. While BIA showed no significant changes in muscle mass, CT analysis revealed increased thigh muscle cross-sectional area, and ^1^H-MRS indicated improvements in intramuscular fat composition. A significant correlation emerged between reduced intramyocellular lipids and improved physical performance measures (*p* < 0.05).

**Conclusion:**

B-SES demonstrated safety and potential efficacy in improving physical function and muscle quality among frail elderly HD patients. From a public health perspective, B-SES may serve as a feasible and accessible intervention for this population, particularly in resource-limited settings. However, further studies are needed to determine its cost-effectiveness in comparison with conventional exercise therapy.

## 1 Introduction

The prevalence of chronic kidney disease (CKD) in Japan continues to rise, particularly among the elderly population. As of 2022, approximately 347,474 patients were receiving hemodialysis (HD) treatment, with 39,683 new cases initiated that year. Notably, patients aged 65 and overrepresented 71.0% of the total HD population (1).

HD patients typically experience reduced physical activity due to treatment-related bed rest and post-dialysis fatigue, resulting in accelerated accumulation of aging-related substances compared to non-HD individuals (2) (3). This sedentary behavior is particularly concerning in elderly HD patients, as it can exacerbate functional decline and increase complication risks (4).

While exercise therapy effectively maintains and improves physical function, adherence remains challenging for elderly HD patients, especially those with frailty (5). Belt-type electrical stimulation (B-SES) has emerged as a promising alternative therapeutic approach (6). Previous research involving younger and middle-aged patients with cardiac conditions and those undergoing HD has demonstrated that B-SES can safely and effectively enhance muscle strength (7). However, evidence regarding its safety and efficacy specifically in frail elderly HD patients remains limited (8).

Body composition assessment plays a crucial role in evaluating nutritional status (9), treatment effectiveness, and prognostic outcomes in elderly HD patients (10). Despite the availability of various measurement techniques, the optimal method for accurate body composition assessment in this population remains unclear. This study is the first to evaluate the effects of B-SES on skeletal muscle mass in frail elderly individuals aged 65 and older, utilizing CT, MRS, and body composition measurements for comparative analysis. This exploratory pilot study had two distinct but complementary aims: The primary objective was to evaluate the safety and feasibility of belt-type electrical stimulation (B-SES) as an alternative intervention to conventional exercise in elderly patients on hemodialysis. The primary outcome was defined as the change in quadriceps muscle cross-sectional area measured by computed tomography (CT), reflecting localized skeletal muscle adaptation. Secondary aims included an exploratory evaluation of the sensitivity and clinical applicability of different body composition measurement methods—CT, bioelectrical impedance analysis (BIA), and proton magnetic resonance spectroscopy (^1^H-MRS)—to inform future clinical research methodologies.

This study had two primary objectives:

First, in this exploratory pilot study, the primary outcome was defined as the change in quadriceps muscle cross-sectional area measured by CT, given its relevance as a direct indicator of skeletal muscle adaptation. Secondary outcomes included physical function tests, biochemical markers, and intramyocellular lipid composition. Second, to investigate the comparative accuracy of different body composition measurement methods and to analyze relationships between skeletal muscle mass and functional measures (grip strength, calf circumference, Timed Up and Go test, and Short Physical Performance Battery) (11).

The findings aim to enhance our understanding of B-SES therapy and to improve the accuracy of body composition assessment in elderly HD patients.

## 2 Materials and methods

### 2.1 Design

**Figure 1:**
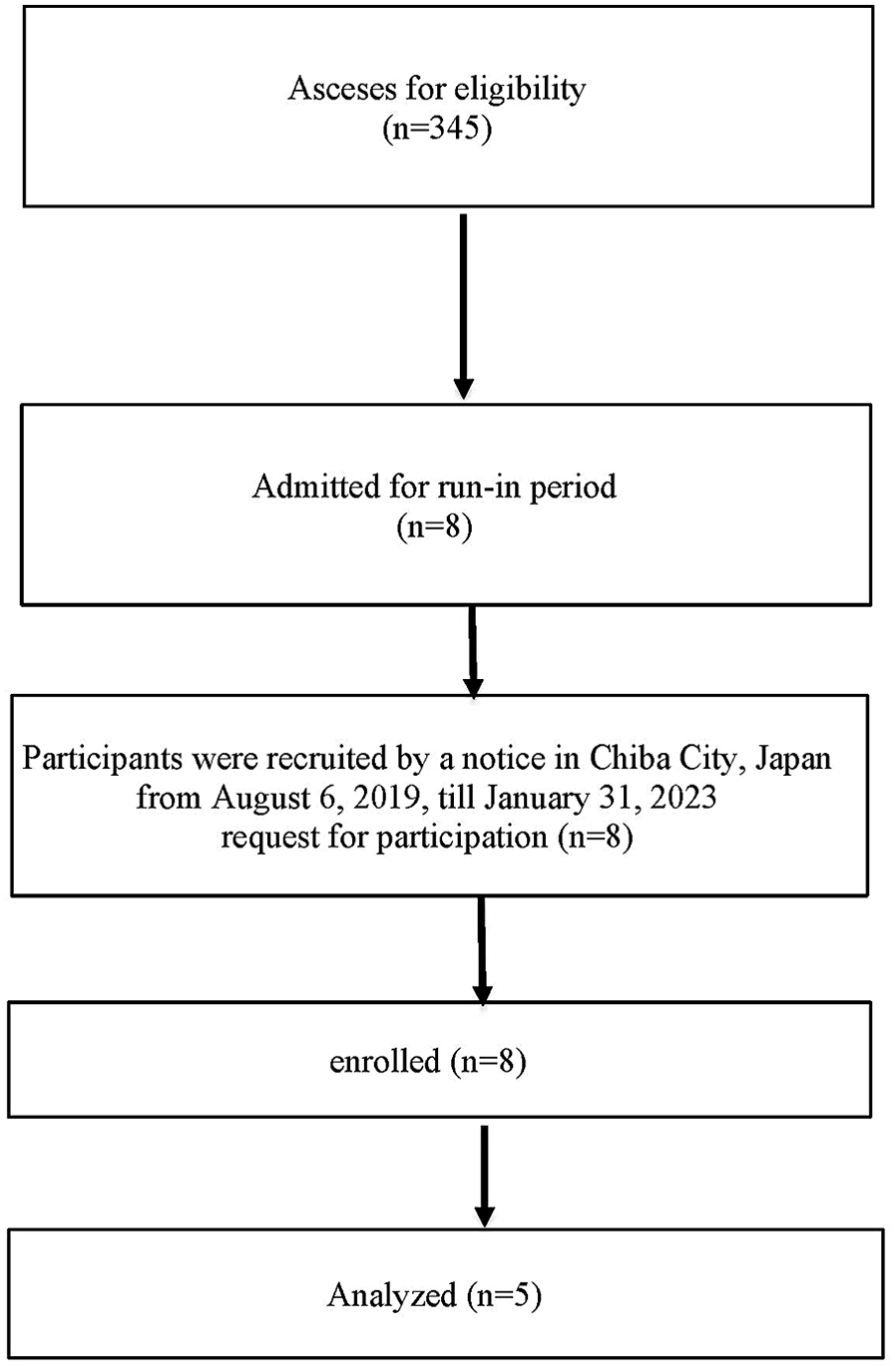
Flow chart enrollment: figure shows the flow of participant recruitment and selection. Participants were recruited from among hemodialysis patients receiving treatment at the cooperating hospital.

This study enrolled elderly end-stage maintenance hemodialysis patients aged 65 and over at a single center in Japan, between August 2019 and January 2023 (Figure 1). This prospective interventional study aimed to evaluate the safety and efficacy of B-SES as an alternative to exercise for frail elderly hemodialysis patients. The evaluation included assessments of physical function, radiological findings, and various biochemical parameters.

Additionally, we investigated differences in skeletal muscle mass measurements across different body composition assessment methods. The relationship between skeletal muscle mass and physical function indicators (grip strength, calf circumference, Timed Up and Go test, and Short Physical Performance Battery) was analyzed to improve the accuracy of body composition assessment in elderly hemodialysis patients.

### 2.2 Participants

The study enrolled a total of 8 participants. The inclusion and exclusion criteria, as well as participant recruitment methods, are outlined below. A flowchart (Figure 1) provides a clear depiction of the participant recruitment process, including the number of participants who were screened, enrolled, and ultimately completed the study. The participants' baseline characteristics are summarized in Table 1.

**Table 1:** Descriptive characteristics of the participants.

Age (years)	Men (%)	BMI (kg/m^2^)	dialysis vintage (month)	dialysis adequacy	Dialysis duration
78.0 ± 1.3	80.0	20.5 ± 1.0	78.0 ± 16.1	1.4 ± 0.3	4–5 h, three times per week

Values are presented as mean ± error; BMI, body mass index; Dialysis adequacy was assessed using Kt/V; Primary diseases (%): Diabetic kidney disease: 40 (%), Chronic glomerulonephritis: 40 (%), Hypertensive nephrosclerosis: 20 (%).

### 2.3 Design

This study was approved by the Ethics Committee of Tsukuba University of Technology (Approval No. 2019-02) and registered with the UMIN Clinical Trial Registration Number: UMIN000057409. All participants were fully informed about the purpose, procedures, and potential risks of the study, and voluntary written informed consent was obtained from each participant. This study was conducted in accordance with the Declaration of Helsinki and ethical guidelines applicable in both national and international contexts.

A total of 8 participants were ultimately enrolled. The inclusion and exclusion criteria for participants are as follows.

Inclusion Criteria: • End-stage kidney disease patients on hemodialysis • Minimum 3 months of hemodialysis treatment • Stable hemodynamics during outpatient maintenance hemodialysis • Age between 65 and 90 years • Daily activity level below 4000 steps (12) • Voluntary consent to participate.

Exclusion Criteria: • Sensory impairment • Walking difficulties • Malignant neoplasms • Severe edema • Skin conditions preventing electrode attachment • Uncontrolled blood pressure (systolic ≥180 mmHg or diastolic ≥110 mmHg) • Concurrent participation in other clinical studies • Acute conditions requiring immediate treatment • Implanted electronic devices • Temporary pacing or intra-aortic balloon pumping therapy • Psychiatric disorders or severe dementia • Other conditions deemed unsuitable by the investigator.

### 2.4 Sample size

Given the exploratory nature of this pilot study investigating B-SES in frail elderly hemodialysis patients, we performed a sample size calculation based on previous electrical stimulation studies in dialysis patients. Using G*Power 3.1.9.4 software, we estimated that 8 participants would be required to detect a moderate effect size (Cohen's d = 0.80) with 80% power at a significance level of *α* = 0.05 for paired comparisons of pre- and post-intervention measurements. This calculation was based on changes in muscle mass and physical function parameters from prior studies utilizing electrical stimulation in dialysis patients.

The final analysis included 5 participants who completed the 12-week intervention, representing a 37.5% dropout rate. While this sample size is smaller than initially planned, it aligns with other pilot studies in this vulnerable population and provides valuable preliminary data for future larger-scale investigations. The reduced sample size limits the statistical power of our analyses, and results should be interpreted as hypothesis-generating rather than conclusive. Future studies should aim for larger sample sizes with appropriate control groups to confirm these preliminary findings.

### 2.5 Evaluation before and after intervention

**Figure 2:**
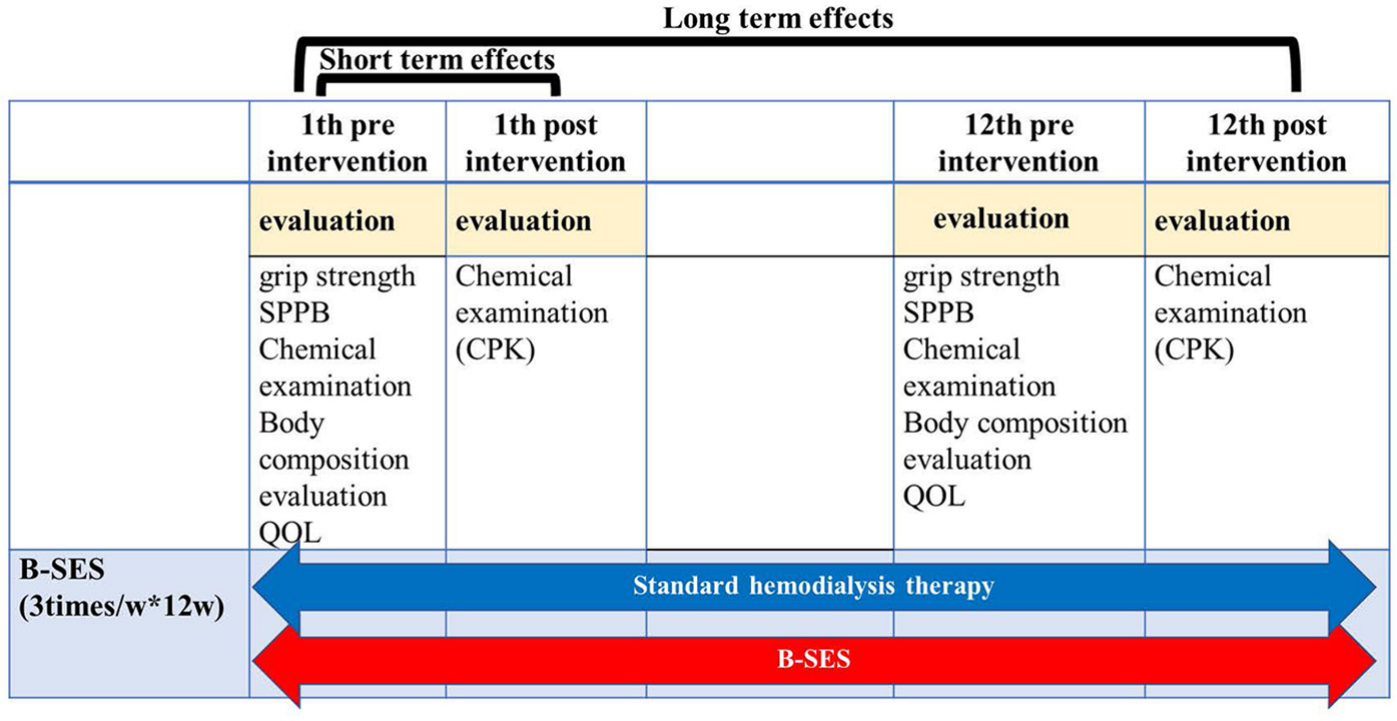
Procedure and measurement setup: figure shows the evaluation included assessments of physical function, radiological findings, and various biochemical parameters. We also investigated differences in skeletal muscle mass measurements among different body composition assessment methods. The relationship between skeletal muscle mass and physical function indicators (grip strength, calf circumference, Timed Up and Go test, and Short Physical Performance Battery) was analyzed to improve the accuracy of body composition assessment in elderly hemodialysis patients.

This study evaluated both acute and chronic effects of B-SES intervention (Figure 2).

Acute Responses: • Biochemical markers (CPK, CRP) • Hemodynamic parameters (blood pressure and heart rate).

Chronic Adaptations: Comprehensive assessments were conducted at baseline and after 12 weeks (Figure 2), including:

1. Physical Function • Muscle strength • Short Physical Performance Battery (SPPB)
2. Biochemical Parameters • CPK, CRP, BUN, IGF-1, IL6, TNF*α*
3. Quality of Life • SF-8 questionnaire
4. Medication Usage
5. Body Composition • Bioelectrical impedance analysis (BIA) • Computed tomography (CT) • 1H-magnetic resonance spectroscopy (1H-MRS)

### 2.6 B-SES protocol and implementation

**Figure 3:**
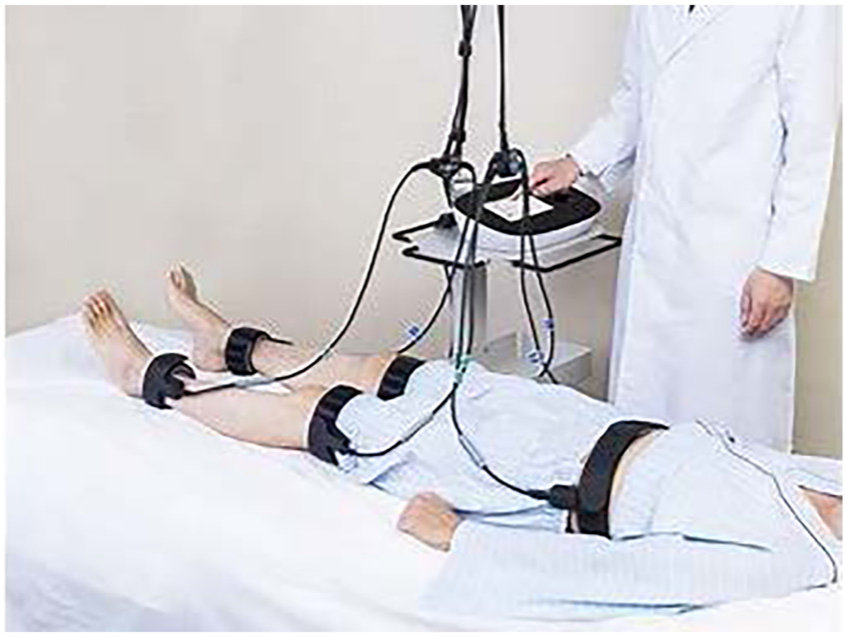
The B-SES intervention was delivered using G-TES (homer Ion institute Co., Ltd., Tokyo, Japan), a specialized therapeutic electrical stimulator: the results from PCS **(A)** and MCS **(B)** did not show any significant differences among the three groups.

The B-SES intervention was delivered using G-TES (Homer Ion Institute Co., Ltd., Tokyo, Japan), a specialized therapeutic electrical stimulator (Figure 3). Treatment parameters were configured as follows:

Technical Parameters: • Frequency: 20 Hz • Duty cycle: 5 s on, 2 s off • Pulse width: 250 *μ*s • Waveform: Exponentially increasing • Duration: 40 min per session (13).

Implementation Protocol: • Frequency: Three sessions per week for 12 weeks (36 total sessions) • Timing: During the first half of hemodialysis treatment • Electrode placement: Five belt-type electrodes positioned on the trunk, bilateral thighs, and lower legs • Intensity: Individually adjusted to maximum tolerable level.

Stimulation Intensity Progression: Thigh Region: • Initial: 1.70/1.79 mA (range: 0.8–2.8 mA) • Final: 3.15/3.47 mA (range: 2.1–7.4 mA).

Lower Leg Region: • Initial: 0.95/0.97 mA (range: 0.4–1.4 mA) • Final: 1.55/1.74 mA (range: 1.1–3.9 mA).

All additional stimulation parameters followed the protocol established by Homma et al. (14). Participants maintained their usual lifestyle throughout the study period, with no specific exercise instructions provided until after the intervention period, when voluntary training guidance was offered.

#### 2.6.1 Physical function

• Muscle strength

Hand-grip strength test was performed as an indicator of overall strength. This test was conducted using a hydraulic hand-held dynamometer (Takai TKK5401, Takei Scientific Instruments Co., Ltd., Tokyo, Japan) with a 0.1 kg accuracy. During the test, subjects kept a standardized position (standing up, with the elbow in full extension) for 2–3 s of maximal pressure. All the subjects repeated the test twice with each hand, alternately. The researchers recorded the best score from the two attempts (15).

• Short Physical Performance Battery (SPPB)

A comprehensive assessment of lower extremity function was performed using the Short Physical Performance Battery (SPPB), according to a previous study (16). The assessment procedure consisted of (1) tandem stand, (2) 4 m walk test, and (3) 5-Time-Sit-To-Stand (5-STS) tests, and the times and scores were recorded. For all measurement elements, the use of knee braces and walking aids was optional.

#### 2.6.2 Biochemical examination (CPK, CRP, BUN, IGF-1, Il6, TNF*α*)

Blood biochemistry tests were performed during the baseline assessment and on the last day of the intervention. Blood biochemistry tests examined creatine kinase (CK), blood urea nitrogen (BUN), C-reactive protein (CRP), interleukin-6 (IL-6), tumor necrosis factor-α (TNF*α*), insulin-like growth factor 1 (IGF-1).

#### 2.6.3 QOL (Sf8)

Health-related QOL (SF-8: The Medical Outcomes Study 8-item Short Form Health Survey) (16) is a short version of SF-36 which measures health-related QOL using 8 subscales. Two summary scores, physical health component summary score (PCS) and mental health component summary score (MCS), are calculated from the 8 subscale scores. A higher score indicates better quality of life. PCS and MCS were measured at baseline and 12 weeks.

#### 2.6.4 Medication usage

Medication profiles were assessed through examination of electronic medical records (EMR) and structured patient interviews. Dialysis patients rely on hospital transportation, and due to their long-established lifestyle habits, they consistently had a daily step count of below 4,000 steps, both before and after the intervention, as measured using a physical activity monitor over one week. Furthermore, there were no changes in medications or dialysis membranes during the study period. Therefore, any changes in physical activity and related outcomes are mainly attributed to the effects of belt-type electrical stimulation.

#### 2.6.5 Body composition

• Bioelectrical impedance analysis (BIA)

BIA were performed on the same day, shortly after the hemodialysis session. Body composition was assessed by a multi-frequency (2.5–350 kHz) body composition analyzer (MLT-550N, SK Medical, Japan), using the wrist–ankle method. The bioimpedance device automatically generated values for extracellular water ratio, skeletal muscle mass, edema ratio, and body fat percentage.

• Computed tomography (CT)

Study participants received volumetric non-contrast enhanced CT scans [Supria Grande, FUJIFILM Medical Systems, Japan] to assess skeletal muscle CSA and volume of tissue (17, 18). Image analysis was performed using Virtual Place New NT software (Canon Medical Systems Corporation, formerly AZE) (19). Scanning parameters were: 64-channel detector with 0.625 mm slice thickness, 120 kV tube voltage, ACE (SD13, 5 mm slice), BP1.1, F32IP3, with mean DLP of 956.2 ± 24.4 mGy·cm. Analysis was conducted on 0.625 mm slice thickness images. Tissue volumes were measured by extracting regions corresponding to predetermined CT number ranges for fat and muscle using the workstation's analytical functions (20).The CT measurements used in this study were not performed for research purposes and did not involve additional radiation exposure. The thigh muscle mass measurements were made using CT images that were originally acquired for osteoporosis screening, and no new radiation exposure was introduced for research purposes.

• ^1^H-magnetic resonance spectroscopy (^1^H-MRS)

^1^H-magnetic resonance spectroscopy (^1^H-MRS) was performed using a 1.5 T MR scanner (Vantage Titan, Canon Medical Systems Corporation, Japan, Version 2.31) (21). All ^1^H-MRS data were processed using LCModel software with DICOM communication “Myrian®” (LA Systems Inc.).

Spectroscopic measurements were acquired using PRESS sequence with the following parameters: TR = 2000ms, TE = 32 ms, voxel size = 2 cm × 2 cm × 2 cm, 128 signal averages, with a total acquisition time of 4:18 min. The concentrations of intramyocellular lipids (IMCL) and extramyocellular lipids (EMCL) were quantified using LCModel analysis software (21, 22).

#### 2.6.6 Statistical analysis

The baseline characteristics were expressed as mean ± standard error. Changes in CPK and CRP levels were analyzed using one-way analysis of variance (ANOVA). Pre- and post-intervention comparisons were performed using either the Wilcoxon signed-rank test for non-parametric data or Student's *t*-test for parametric data, depending on the normality of distribution. The handling of missing values followed procedures established in the literature (23). Statistical significance was set at *p* < 0.05. All statistical analyses were performed using SPSS version 27.0 (IBM Corp., Armonk, NY, USA).

For paired comparisons between baseline and post-intervention measurements, were analyzed using paired Student's *t*-test were employed to statistically analyze the logarithmically transformed wear data using the Wilcoxon signed-rank test, and Spearman's product-moment correlation coefficient was used for parameters of various measurements of skeletal muscle mass and other parameters.

## 3 Results

Three participants were excluded for the following reasons:

Medical reasons (*n* = 1), Refusal to participate (*n* = 2).

As a result, a total of 5 participants completed the intervention.

### 3.1 Safety and adherence

The 12-week intervention was completed by five out of eight participants (62.5% completion rate). Three participants withdrew: two due to decreased motivation and one due to hospitalization for pneumonia unrelated to the intervention. No changes in medication regimens were required during the study period, supporting the safety profile of B-SES.

### 3.2 Physical function and inflammatory markers

While grip strength and calf circumference remained stable, notable trends toward improvement were observed in functional mobility measures:

• Timed Up and Go (TUG) test performance improved (specific values and p-value)
• Short Physical Performance Battery (SPPB) scores increased (specific values and *p*-value)
• Importantly, these improvements occurred without an elevation in inflammatory markers.

### 3.3 Body composition analysis

**Figure 4:**
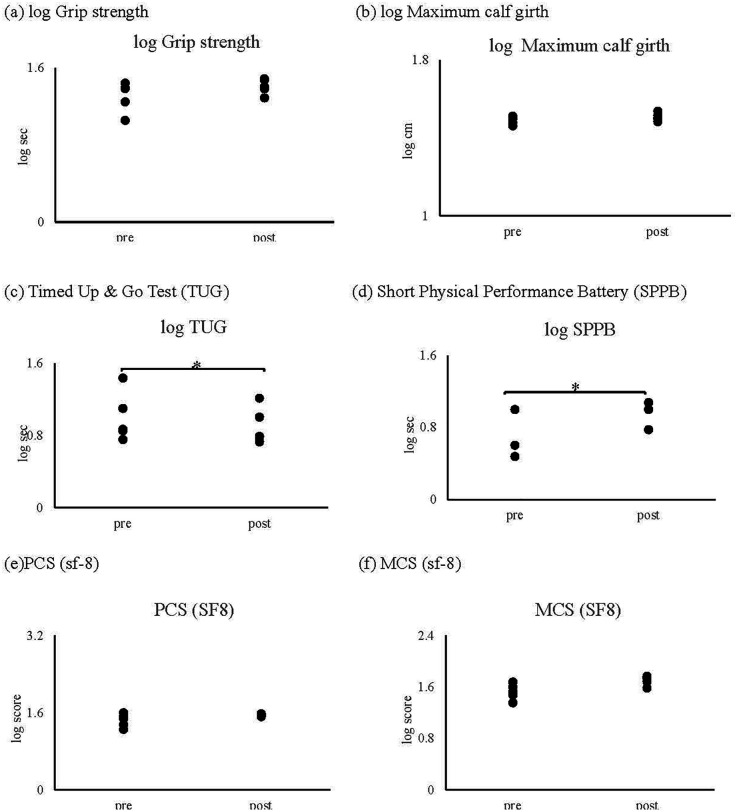
Result of physical performance: as shown in figure, no significant changes were observed in **(a)** grip strength and **(b)** maximum calf circumference following the intervention. However, significant improvements were demonstrated in both **(c)** Timed Up and Go (TUG) test performance and **(d)** Short Physical Performance Battery (SPPB) scores (*p* = 0.043). Quality of life measures, including **(e)** Physical Component Summary (PCS) and **(f)** Mental Component Summary (MCS) scores of the SF-8, showed no significant changes throughout the intervention period (*p* > 0.1).

Multiple imaging modalities revealed differential findings:

• Bioelectrical impedance analysis (BIA) showed no significant changes in muscle mass
• Computed tomography (CT) analysis demonstrated increased thigh skeletal muscle cross-sectional area (specific values and *p*-value)
• Proton magnetic resonance spectroscopy (^1^H-MRS) indicated improvements in intramuscular fat composition (Figure 4c)

### 3.4 Correlational analyses

**Figure 5:**
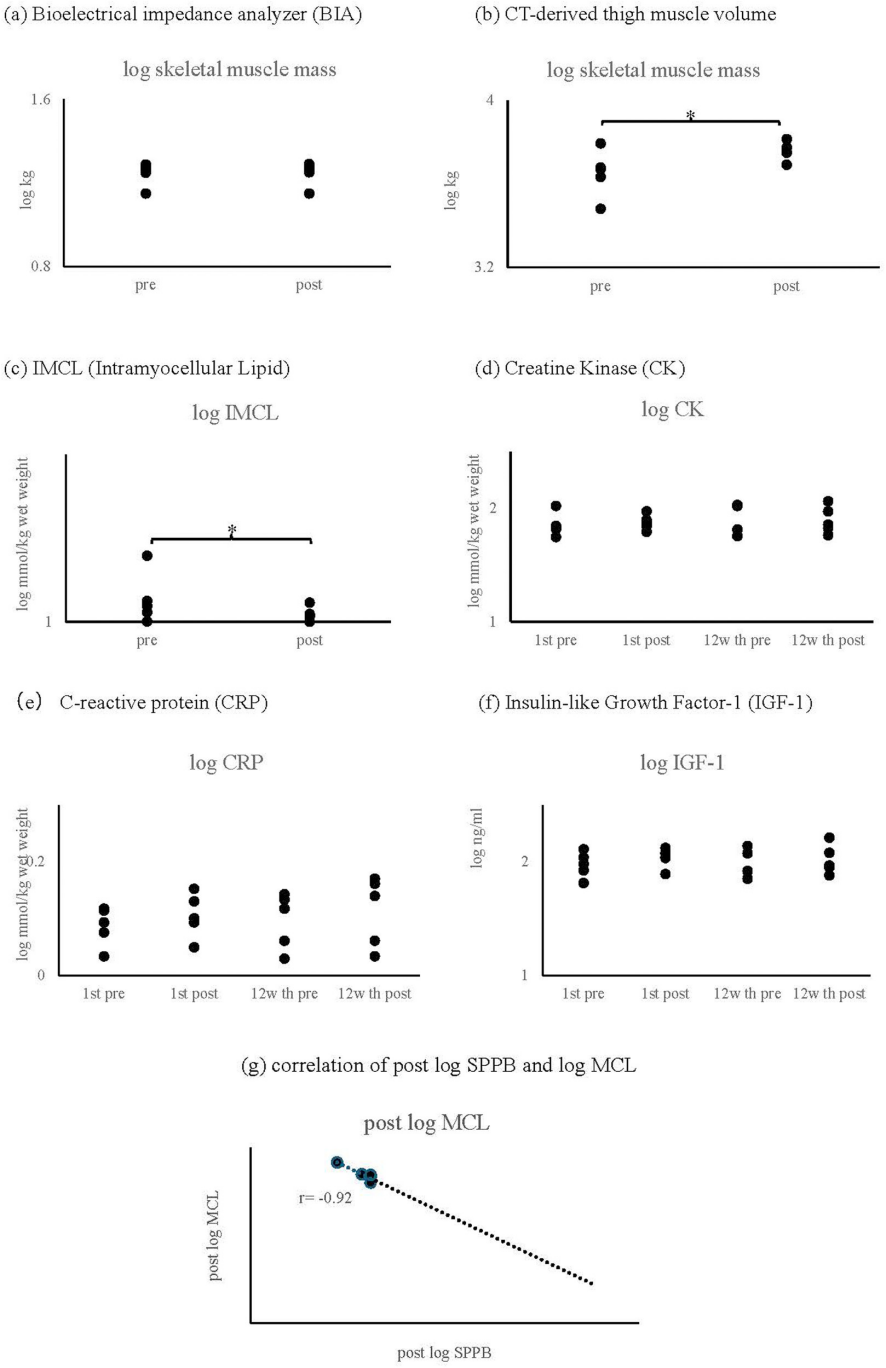
Result of biochemical and body composition parameters. **(a)** Skeletal muscle mass measured by bioelectrical impedance analysis (BIA). **(b)** Thigh skeletal muscle volume assessed by computed tomography (CT). **(c)** Intramyocellular lipid (IMCL) content measured by proton magnetic resonance spectroscopy (¹H-MRS). **(d)** Creatine phosphokinase (CPK). **(e)** C-reactive protein (CRP). **(f)** Insulin-like growth factor-1 (IGF-1). **(g)** Correlation between IMCL content and SPPB score (*r* = −0.92, *p* = 0.019).

A strong negative correlation (*r* = −0.92, *p* = 0.019) was observed between intramyocellular lipids measured by ^1^H-MRS and SPPB scores (Figure 5g). No significant correlations were found between physical function measures and other body composition parameters (skeletal muscle mass, body fat mass, or body fat percentage).

### 3.5 Quality of life

Analysis of SF-8 components revealed no significant changes throughout the intervention period (all *p* > 0.05; Figures 4e,f).

## 4 Discussion

This study was designed as an exploratory pilot trial with hypothesis-generating intent rather than hypothesis-testing. Given the limited sample size, the study was not powered to detect definitive clinical effects, but rather aimed to provide initial evidence regarding the feasibility, safety, and potential efficacy of B-SES in a frail elderly dialysis population. The findings are presented as preliminary trends to inform the design of larger, controlled trials. This is the first study to compare and analyze the effects of B-SES on skeletal muscle mass in frail elderly individuals aged 65 and older using CT, MRS, and body composition measurements. To evaluate B-SES as an alternative to exercise, we studied eight maintenance dialysis patients aged 65 and older with CKD stage 5D. Although three participants withdrew for reasons unrelated to the intervention during the 12-week B-SES therapy period, the treatment was safely administered to frail elderly patients with chronic renal failure, with no observations of sudden blood pressure drops or musculoskeletal injuries. Biochemical analysis showed no immediate or long-term changes in creatine phosphokinase (CPK) levels over the 12-week period, suggesting minimal risk of muscle damage. In this study, we observed a decrease in TNF-α following electrical stimulation, like the anti-inflammatory effects reported with regular exercise (24, 25). This suggests that electrical stimulation may have exercise-like anti-inflammatory effects. Furthermore, no changes were observed in CK, CRP, and IL-6 levels. These findings suggest that electrical stimulation can suppress TNF*α* expression even at sub-threshold intensities for muscle damage.

Ectopic fat accumulation in skeletal muscle, particularly IMCL, has been associated with sarcopenia, reduced muscle strength, and increased cardiovascular disease risk. Our study observed preliminary reductions in IMCL and improvement trends in physical function indicators (TUG and SPPB) after the 12-week intervention. These findings, consistent with previous research, suggest improvements through enhanced IMCL metabolism via skeletal muscle activation.

Despite the small sample size, physical function assessments showed improvement trends in SPPB sub-items, including walking speed and chair-stand performance, after 12 weeks of B-SES intervention. Although physical function improved, no significant changes were observed in SF-8 scores. This may be attributable to the short duration of the intervention and the unique characteristics of the study population. Elderly patients on maintenance hemodialysis often have relatively stable disease courses and receive thrice-weekly, four-hour dialysis sessions via hospital transportation. Improvements in perceived quality of life may require a longer intervention period and more sustained changes in daily activities to become evident. These results align with previous studies using electrode pad electrical stimulation. Notably, patients with more severe standing and walking disabilities showed improved balance ability and walking speed post-intervention. While dry weight remained unchanged, intramuscular fat, which was not clearly detected by BIA, showed improvement trends, and CT scans revealed significant increases in thigh muscle mass. These discrepancies may be explained by the methodological characteristics of each modality. CT is highly sensitive to regional muscle mass changes and can provide precise cross-sectional imaging of targeted anatomical sites. In contrast, BIA estimates total body composition based on impedance, and its accuracy may be limited in older hemodialysis patients due to altered hydration status and fluid retention. Given that BIA may overestimate muscle mass by interpreting extracellular fluid as lean mass, it may not be optimal for assessing localized muscle changes in this population. Thus, CT may be more appropriate than BIA for evaluating localized muscle changes in frail elderly dialysis patients.

These observations suggest that B-SES may improve basic motor abilities through qualitative changes in lower limb muscles among frail elderly individuals unable to maintain regular exercise routines. Although there was no significant change in grip strength, an increase in muscle mass measured by CT and a reduction in IMCL assessed by ^1^H-MRS were observed. These preliminary findings suggest that B-SES may improve basic motor abilities by enhancing both localized muscle strength and related motor functions, particularly through broader stimulation than electrode pad systems. The results indicate that B-SES, which provides broader stimulation than electrode pad systems, may contribute to improvements in both localized muscle strength and related motor functions. Sample size calculations for pilot studies often focus on feasibility and safety outcomes rather than efficacy endpoints (26), and our sample size, though small, allowed for meaningful assessment of these primary objectives (27). Therefore, further studies with larger participant numbers and control group comparisons are needed.

From public health and universal health coverage perspectives, B-SES may represent a feasible and accessible intervention for frail elderly HD patients. However, the cost-effectiveness of this approach remains to be evaluated through formal economic analyses and comparative studies with standard rehabilitation strategies. Nevertheless, considering Japan's aging population and increasing HD patient numbers, implementing B-SES therapy could potentially improve patient outcomes while alleviating strain on healthcare systems. This intervention may be particularly valuable in resource-limited settings or where access to conventional exercise therapy is challenging. Larger-scale studies are necessary to establish the cost-effectiveness and feasibility of incorporating B-SES therapy as a standard care component for frail elderly HD patients.

## 5 Study limitations and future directions

This study has several important limitations that should be considered. First, the small sample size (*n* = 5 completing the intervention) limits the statistical power and generalizability of our findings. While our sample size calculation was appropriate for a pilot study focusing on safety and feasibility (20), larger studies are needed to confirm efficacy outcomes. Second, the lack of a control group makes it difficult to distinguish intervention effects from natural time course changes. Third, the 12-week intervention period may be insufficient to observe long-term adaptations in muscle mass and function. Additionally, the single-center design and specific geographical location (Japan) may limit the external validity of our findings to other populations. The preliminary findings in TUG, SPPB, CT-derived muscle mass, and IMCL are promising; however, future randomized controlled trials are needed to establish causality. The study also lacked the assessment of potentially important outcomes, such as muscle protein synthesis markers and detailed analysis of physical activity patterns outside the intervention sessions.

Future studies should address these limitations through:

Larger Scale Trials.

Multi-center randomized controlled trials with adequate sample sizes (estimated *n* = 60–80 based on our pilot data).

Inclusion of appropriate control groups (sham stimulation or standard care).

Longer intervention periods (6–12 months) with follow-up assessments.

Comprehensive Outcome Assessment.

Addition of muscle biopsy analysis for molecular mechanisms.

Detailed physical activity monitoring using wearable devices.

Assessment of healthcare utilization and cost-effectiveness.

Quality of life measures specific to dialysis patients.

Implementation Research.

Investigation of factors affecting adherence and compliance.

Development of optimal stimulation protocols for different patient subgroups.

Evaluation of B-SES integration into routine clinical care.

Regarding sample size calculation for this pilot study, we followed recommendations for pilot studies in rehabilitation research (21), which suggest that 8–10 participants are typically sufficient to assess safety, feasibility, and preliminary efficacy. Our initial sample size of 8 participants was determined based on:

Effect sizes from previous electrical stimulation studies in younger dialysis patients.

Practical considerations regarding recruitment from the eligible population.

The primary focus on safety and feasibility outcomes rather than efficacy endpoints.

While the final sample of 5 participants was smaller than planned, it still provided valuable information about safety and potential efficacy, guiding future larger-scale investigations. Given the absence of a comparison group, definitive conclusions about the efficacy of B-SES cannot be drawn from this study. Future randomized controlled trials are required to validate these preliminary findings. These findings will inform more precise sample size calculations for subsequent randomized controlled trials.

## Data Availability

The datasets presented in this study can be found in online repositories. The names of the repository/repositories and accession number(s) can be found in the article/Supplementary Material.
